# An Optimized Adsorbent Sampling Combined to Thermal Desorption GC-MS Method for Trimethylsilanol in Industrial Environments

**DOI:** 10.1155/2012/690356

**Published:** 2012-08-23

**Authors:** Jae Hwan Lee, Chunrong Jia, Yong Doo Kim, Hong Hyun Kim, Tien Thang Pham, Young Seok Choi, Young Un Seo, Ike Woo Lee

**Affiliations:** ^1^Headquarters of GemVax & KAEL Co., Ltd., 894 Tamnip-dong, Yuseong-gu, Daejeon 305-510, Republic of Korea; ^2^R&D Institute of GemVax & KAEL Co., Ltd., 894 Tamnip-dong, Yuseong-gu, Daejeon 305-510, Republic of Korea; ^3^School of Public Health, University of Memphis, 337 Robison Hall, Memphis, TN 38152, USA; ^4^Center for Gas Analysis, Division of Metrology for Quality of Life, Korea Research Institute of Standards and Science, 1 Doryong-dong, Yuseong-gu, Daejeon 305-340, Republic of Korea

## Abstract

Trimethylsilanol (TMSOH) can cause damage to surfaces of scanner lenses in the semiconductor industry, and there is a critical need to measure and control airborne TMSOH concentrations. This study develops a thermal desorption (TD)-gas chromatography (GC)-mass spectrometry (MS) method for measuring trace-level TMSOH in occupational indoor air. Laboratory method optimization obtained best performance when using dual-bed tube configuration (100 mg of Tenax TA followed by 100 mg of Carboxen 569), n-decane as a solvent, and a TD temperature of 300°C. The optimized method demonstrated high recovery (87%), satisfactory precision (<15% for spiked amounts exceeding 1 ng), good linearity (*R* ^2^ = 0.9999), a wide dynamic mass range (up to 500 ng), low method detection limit (2.8 ng m^−3^ for a 20-L sample), and negligible losses for 3-4-day storage. The field study showed performance comparable to that in laboratory and yielded first measurements of TMSOH, ranging from 1.02 to 27.30 *μ*g/m^3^, in the semiconductor industry. We suggested future development of real-time monitoring techniques for TMSOH and other siloxanes for better maintenance and control of scanner lens in semiconductor wafer manufacturing.

## 1. Introduction

Trimethylsilanol (TMSOH, CAS No. 1066-40-6) in industrial sectors has gained wide attention due to the widespread use of silicon materials and their detrimental effects on equipments and products [1]. TMSOH is a silanol but often is considered to belong to the siloxane group. It is the most volatile siloxane with a vapor pressure of 73.9 mmHg at 25°C [2]. Siloxanes are a family of silicon containing organic compounds that are widely used in manufacture of commercial and consumer products, for example, detergents, deodorants, and cosmetics [3, 4]. Siloxanes are considered safe to the general population and available toxicological studies target octamethylcyclotetrasiloxane (D4), decamethylcyclopentasiloxane (D5), and dodecamethylcyclohexasiloxane (D6); thus, no inhalation toxicity data are available for TMSOH. Limited oral and skin exposure studies show that TMSOH causes nervous system depression and anesthesia at high doses [5]. Oral toxicity tests determined a no observable effects limit of 160 mg/kg/day in rats [6]. The U.S. Occupational Safety & Health Administration has not set exposure limits for TMSOH [7]. The U.S. National Academies have set 65 mg/m^3^ and 32 mg/m^3^ as 24-hour and long-term spacecraft maximum allowable concentrations for TMSOH, respectively [5]. The U.S. Environmental Protection Agency (EPA) is proposing a chemical action plan for siloxanes to understand human health risk associated with siloxane exposure [8].

The nonhealth hazards from siloxanes use are of more concern in industrial processes. Most widely, the concern arises from siloxanes in biogas emitting from landfills and wastewater treatment [9]. Combustion of siloxane-containing biogas forms silicate particles that may cause severe abrasion of combustion engines and decrease in the efficiency of the equipment [10]. Recently, TMSOH gains special attention in the rapidly growing semiconductor industry. During the ultraviolet lithographic process of the wafer production, TMSOH forms in the reaction between a wafer treatment agent hexamethyldisilazane and water vapor [11]:

(1) $$\begin{array}{c} {\left({\left(\text{C}{\text{H}}_{\text{3}}\right)}_{\text{3}}\text{Si}\right)}_{2}\text{NH}+\text{2}{\text{H}}_{\text{2}}\text{O}\to \text{2}{\left(\text{C}{\text{H}}_{\text{3}}\right)}_{\text{3}}\text{SiOH}+\text{N}{\text{H}}_{\text{3}}. \end{array}$$

Even trace levels of TMSOH can accumulate and form salts on surfaces of scanner lenses over time. The scanner lens is an expensive key device in the semiconductor wafer production line, and TMSOH salts can cause severe and sometimes irreversible damage [11]. Therefore, accurate analysis of airborne TMSOH is required to obtain the best lens performance in the semiconductor manufacturing environments.

Analytical methods for siloxanes often focus on D4–D6 cyclical siloxanes and linear siloxanes, including hexamethyldisiloxane (L2), octamethyltrisiloxane (L3), and decamethyltetrasiloxane (L4), without considering TMSOH [12]. The U.S. EPA's reference method uses evacuated canisters to collect whole air samples of volatile organic compounds (VOCs) [13]; however, recovery of polar compounds like TMSOH in canisters can be problematic [14]. Several studies have reported sampling siloxanes using canisters [15, 16], Tedlar bags [17, 18], and active carbon [19], but they targeted high-concentration siloxanes in biogas, and the methods were not optimized for TMSOH or even ignored TMSOH. Adsorbent enrichment followed by thermal desorption offers a number of advantages over competing methods [20]. Many adsorbents and sampling devices are available, including tubes, badges, and cartridges [21, 22]. Typically, sampling devices are very small, simplifying collection, transport, and storage. Cleaning sorbents is much easier and less expensive than cleaning canisters. Thermal desorption offers high sensitivity, high recovery, and simple operations [23]. Adsorbent sampling has several potential disadvantages, including breakthrough on sampling, a need to match the sorbent to target compounds, the possibility of adsorbent artifacts during sampling (e.g., with exposure to ozone [24]) and thermal desorption, and only single sample analysis after sample collection, compared to multiple analyses possible using whole air sampling. A few studies have applied this technique to measure siloxanes, typically along with other VOCs but often excluding TMSOH [12]. Even a thorough literature review identifies few studies that validate an adsorbent sampling-thermal desorption method for TMSOH [25].

High-sensitivity methods have not been designed specifically for airborne TMSOH in industrial environments that have special needs to control TMSOH, for example, the semiconductor manufacturing workshops. This study develops a thermal desorption (TD)-gas chromatography (GC)-spectrometry (MS) method for trace-level TMSOH analysis in occupational indoor air. The method is optimized by comparing adsorbent configurations and analysis solvents, choosing an appropriate desorption temperature and applying a more sensitive selected ion monitoring (SIM) mode in MS. The method performance is evaluated in laboratory and in a semiconductor fabrication factory.

## 2. Experimental

### 2.1. Reagents, Adsorbents, and Thermal Desorption Tubes

Trimethylsilanol (98.5%) was obtained from Apollo Scientific Ltd., Cheshire, UK; methanol (99.9%) from Duksan, Ansan, Republic of Korea; n-pentane (99%) from Junsei, Tokyo, Japan; n-hexane (96%) from Kanto Chemical, Tokyo, Japan; n-decane (99.5%) from Dae Jung, Siheung, Republic of Korea. Tenax TA (poly(2,6-diphenyl-p-phenylene) oxide, 60–80 mesh, 35 m^2^/g, for C_5_–C_26_ VOCs), Carbopack B (graphitized carbon black, 60–80 mesh, 100 m^2^/g, for C_5_–C_12_ VOCs), Carbopack C (graphitized carbon black, 60–80 mesh, 10 m^2^/g, for C_12_–C_20_ VOCs), Carboxen 569 (carbon molecular sieve, 20–45 mesh, 485 m^2^/g, for C_2_–C_5_ VOCs), and Carbosieve-SIII (carbon molecular sieve, 60–80 mesh, 820 m^2^/g, for C_2_–C_5_ VOCs) were obtained from Supelco, Bellefonte, PA, USA. Empty glass thermal desorption tubes (18 cm long, 4 mm I.D.) were obtained from Gerstel, Mülheim, Germany.

### 2.2. Configurations of Adsorbent Tubes

To determine the adsorbent or adsorbent combination that has the highest recovery, we prepared three sets of adsorbent tubes based on adsorbents' affinity abilities: (1) single-bed tubes, packed with 180 mg of Tenax TA; (2) dual-bed tubes, packed with 100 mg of Tenax TA at the sampling side, followed by 100 mg of Carboxen 569; (3) triple-bed tubes, packed with 125 mg of Carbopack C, 175 mg of Carbopack B, and 123 mg of Carbosieve-SIII. The triple-bed tube was packed to test if Carbosieve-SIII, a stronger adsorbent, had better performance. Adsorbent beds were retained or separated by nonsilanized glass wool (Supelco, St. Louis, MO, U.S.A.) plugs. Before use, tubes were conditioned at 300°C in a Gerstel tube conditioner (Gerstel, Mülheim, Germany) with an 80 mL/min of ultrahigh-purity (UHP) nitrogen (Korea Noble Gas Co., Daejeon, Republic of Korea) flow for 6 hours. The flow direction was opposite to the sampling direction to more effectively remove contaminants. After conditioning, clean tubes were sealed with PTFE caps and kept at 4°C in a refrigerator.

### 2.3. Development of the TD-GC-MS Method

#### 2.3.1. Tube Spiking Procedure

Clean tubes were removed from the refrigerated storage container and equilibrated at room temperature before use. An adsorbent tube was then spiked with 1 *μ*L aliquot of solution through a lab-made injector being flushed with 100 mL/min of UHP nitrogen for 1 min. This purging process effectively transferred the TMSOH solutions into the tube.

#### 2.3.2. Selection of Solvents

An appropriate solvent is critical to GC-MS analysis as the solvent is the carrier of analytes. Methanol is a widely used solvent for VOC analysis because of its fast elution, significant low background, non-aggressive behavior into GC column, and acceptable level of compound solvatation, as well as easy separation with other chemicals. However, preliminary tests showed that methanol had detrimental effects on TMSOH separation, including formation of artifacts from chemical reactions. Thus, four candidate solvents, methanol, n-pentane, n-hexane, and n-decane, were tested to obtain the best separation. Standard TMSOH solution was diluted in these solvents to form 200 *μ*g/mL of each solution. Dual-bed tubes were spiked with 1 *μ*L of each solution and then underwent the same TD-GC-MS analysis. At least three tests were performed for each solvent.

#### 2.3.3. Selection of Desorption Temperature

Dual-bed tubes were spiked with 100 or 200 ng of TMSOH in n-decane, and then thermally desorbed at 150, 200, 250, and 300°C, respectively. Duplicate samples were used for each test.

#### 2.3.4. Selection of Adsorbent Configurations

For the three-tube configurations, each was spiked with 200 ng of TMSOH in n-decane and then analyzed following the same TD-GC-MS procedure. The triple-bed tubes contained high surface area (820 m^2^/g) Carbosieve-SIII, which may retain VOCs after the thermal desorption, a phenomenon called the memory effect. To examine this effect, triple-bed tubes were analyzed again after the first thermal desorption. Tests were repeated three times for each tube configuration.

#### 2.3.5. Optimized TD-GC-MS Conditions

Adsorbent tube samples were analyzed on a thermal desorption system (TDS, Gerstel, Mülheim an der Ruhr, Germany) followed by GC-MS (Agilent 7890A/5975C, Agilent Technologies, Santa Clara, CA, U.S.A.). The TDS was mounted on top of a cooled injection system that was used as a cryotrap. TMSOH and other VOCs were cryofocused and concentrated at −30°C using liquid nitrogen, after which they were transferred to the capillary column without discrimination or loss of analytes. The TD-GC-MS conditions (Table 1), based on results of experiments described earlier, were optimized for analyzing TMSOH along with a wide range of C_4_–C_28_ VOCs. The Agilent 5975C MS has synchronous Scan/SIM functionality, meaning that the MS can capture full-scan data and SIM (selected-ion monitoring) data in the same acquisition. Thus, TMSOH was analyzed in the SIM mode with the target ion 75 m/z and two qualifier ions 45 and 47 m/z, which are the three highest ions obtained on the mass spectrum. The SIM mode would significantly increase the sensitivity, ideal for possible low concentrations in air [26]. The full-scan mode could be used to qualify and quantify other VOCs and to identify unknown compounds. TMSOH and other VOCs were identified using NIST05.L Spectral Library (National Institute of Standards and Technology, Gaithersburg, MD, U.S.A.).

### 2.4. Laboratory Performance Evaluation

The laboratory performance experiments were aimed to determine recovery, establish calibrations, check instrument linearity, and determine analysis precision, method detection limits (MDLs), adsorbent retainability, and storage stability. The performance evaluation procedure generally followed the U.S. EPA's guidelines for analyzing VOCs, for example, TO-15 [27] and TO-17 [28].

#### 2.4.1. Recovery

The recovery was calculated as the ratio of abundance for a given amount of TMSOH from TD-GC-MS to that generated from direct injection of the same amount of TMSOH followed by the same GC-MS analysis. The fraction reflects the combined efficiency of adsorption of gaseous compounds and thermal desorption. Recovery experiments were conducted for a wide range of spiked amounts, including 5, 20, 100, and 200 ng. Recovery tests also included redesorption of the tube to check the memory effect. All tests used duplicates. As an indicator of accuracy, recovery is expected to be within ±30% of the true amount [28].

#### 2.4.2. Calibration and Linearity

The initial 7-point calibration was established using loadings of 0.1, 0.5, 1.0, 5.0, 20, 100, and 500 ng, respectively. Calibration solutions were prepared by diluting pure TMSOH to 100 mL of high-purity n-decane. This resulted in series solutions of 0.1, 0.5, 1, 5, 20, 100, and 500 *μ*g/mL, respectively. All the calibration solutions also contained 10 *μ*g/mL of fluorobenzene, a compound used as an internal standard (IS). The target and qualifier ions of fluorobenene were 96 m/z and 70 m/z, respectively. The linear calibration curve was determined by regressing abundance ratio of analyte to IS against mass ratio of analyte to IS. The linearity was evaluated using the *R* ^2^ of the regression. The linearity was also evaluated using *R* ^2^ of the regression line established from log-transformed abundances and amounts. This transformation avoided the inflation of *R* ^2^ caused by high concentrations. All levels ran duplicate samples, with another purpose of determining replicate precision.

#### 2.4.3. Precision

Precision is commonly expressed as relative standard deviation (RSD) for multiple replicates or percent difference (% *D*) between duplicates [29]:

(2) $$\begin{array}{c} \%\;D=\frac{\left|\text{Measurement 1}-\text{Measurement}\;2\right|}{\text{Average of measurements}}\times 100\%, \end{array}$$

where measurement could be abundance, mass, or concentration. The criterion is within 20% for solid adsorbent sampling, but could be lenient, for example, 50% for very low concentrations [26, 28].

#### 2.4.4. Method Detection Limit (MDL)

The MDL was determined by analyzing 7 replicate tubes spiked with a low concentration of TMSOH that was expected to be near the MDL to avoid an artificially high MDL [30]. The MDL was then computed as the product of the standard deviation (SD) for the 7 replicate concentrations and 3.14 (the Student's *t*-value for one-sided 99% confidence for 7 values), that is,

(3) $$\begin{array}{c} \text{MDL}=\text{SD}\times t_{1-\alpha }\left(n-1\right). \end{array}$$

#### 2.4.5. Retainability of TMSOH on Adsorbent(s)

These experiments were aimed to test how well the single- and dual-bed tubes could retain TMSOH. For each tube configuration, three tubes were connected in series, and the first tube was spiked with 200 ng of TMSOH. Then a 100 mL/min flow of N_2_ gas or air was pulled through the tube series for 10, 50, 100, and 200 min, respectively, corresponding to total volumes of 1, 5, 10 and 20 L, respectively. The same procedures were repeated for 5 ng loadings. In each test, amounts of TMSOH obtained from analysis of the front, 1st backup, and 2nd backup tubes were calculated as the percentages of the initial amount spiked to the front tube.

#### 2.4.6. Storage Stability

In this experiment, 10 dual-bed tubes were initially spiked with 10 ng of TMSOH each. Then duplicate tubes were analyzed immediately, and 1, 3, 7, and 14 days after the initial loading, respectively. Tubes were sealed and stored at 4°C in a VOC-free refrigerator, and an internal standard solution was loaded to each tube right before GC-MS analysis. Using the mean of duplicates, storage stability was expressed as the percentage of the initial measurement.

### 2.5. Field Study

The field monitoring was conducted in a wafer manufacturing workshop of a semiconductor fabrication factory in Cheong-Ju City, Republic of Korea, every two weeks from June to October, 2010. Samples were collected at a flow rate of 100 mL/min for 60 or 200 min using a microprocessor-controlled air sampling pump (SIBATA Mini-pump, Σ30, Japan). The initial intention was to measure TMSOH concentrations as well as to compare two tube configurations. Thus, each sampling event used a dual-bed tube and a single-bed tube, and samples were collected side-by-side. Single-bed Tenax tubes showed poor performance as observed in laboratory, so only results from dual-bed tubes were reported. A follow-up field sampling was conducted in the same workshop in August 2011. This sampling collected duplicate 6 L samples and distributed volume (6 L and 20 L) samples. Distributed volume samples are two samples with different volumes in parallel at the same monitoring location. The U.S. EPA recommends this strategy for adsorbent sampling to increase method sensitivity as well as to check reproducibility [28].

### 2.6. Quality Control

Contamination is almost unavoidable in siloxane analysis given many silicon-containing materials used in GC parts; however, the artifacts of concern are cyclic siloxanes [25]. The laboratory performance tests included analyses of solvent and tube blanks. TMSOH was not detected in lab blanks, though other siloxanes were found at trace levels. Clean tubes and field samples were sealed and stored in sealed plastic tubes at 4°C in a VOC-free refrigerator dedicated to tube storage. The sampling flow rate was measured at the beginning and end of the sampling period using an Agilent ADM-3000 digital flowmeter (Agilent Technologies, Santa Clara, CA, U.S.A.). After collection, all samples were shipped to the laboratory and were analyzed within 12 hours to avoid storage loss. Each field sampling used a field blank, and no TMSOH was detected in blank samples in either scan or SIM modes. The calibration curve was updated right before the analyses of each batch of field samples following the same procedure as described earlier.

## 3. Results and Discussion

### 3.1. Method Optimization

#### 3.1.1. Solvent Effects on GC Separation of TMSOH

**Figure 1:**
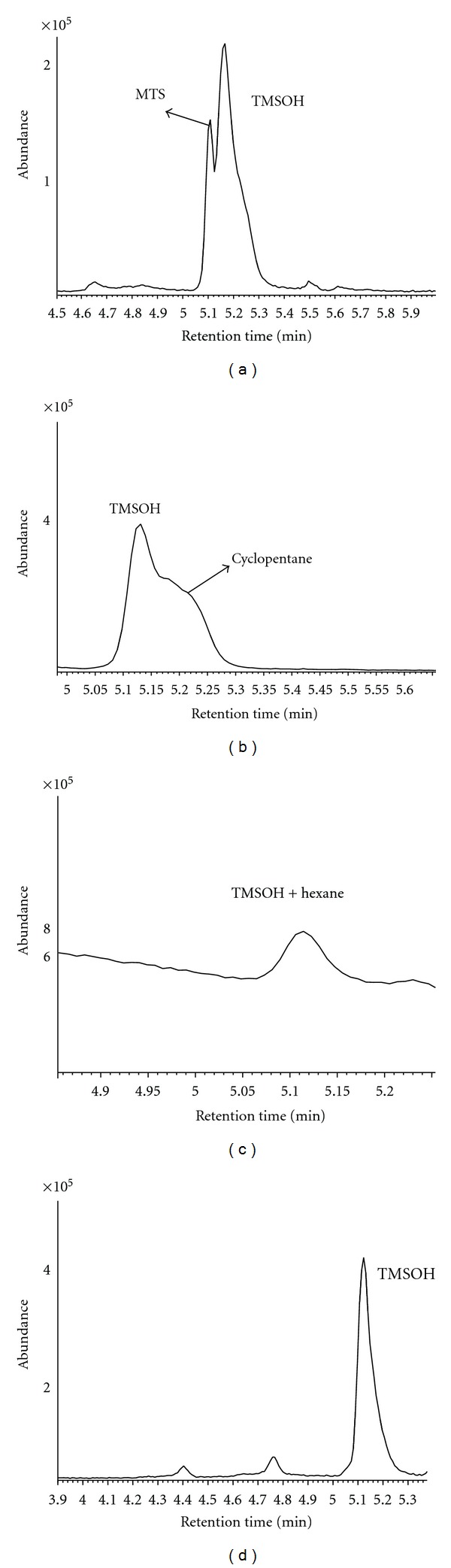
Solvent effects on TMSOH separation. (a) methanol, (b) n-pentane, (c) n-hexane, and (d) n-decane. MTS: methoxytrimethylsilane. Samples were analyzed in scan mode.

Column separation of TMSOH using different solvents is displayed in Figure 1. Repeated tests showed consistent chromatograms for each solvent. Of the four solvents, n-decane displayed the best separation ability, forming a well-separated, sharp, near-symmetric peak of TMSOH (Figure 1(d)). Methanol showed an acid-base reaction with TMSOH in the column:

(4) $$\begin{array}{c} {\text{(C}{\text{H}}_{\text{3}}\text{)}}_{\text{3}}\text{SiOH}+{\text{CH}}_{\text{3}}\text{OH}\to {\left({\text{CH}}_{\text{3}}\right)}_{\text{3}}{\text{SiOCH}}_{\text{3}}+{\text{H}}_{\text{2}}\text{O}, \end{array}$$

where TMSOH was considered a weak base and methanol a weak acid. The reaction formed an artifact methoxytrimethylsilane (MTS, Figure 1(a)), which partially co-eluted with TMSOH. When n-pentane was used, TMSOH co-eluted with cyclopentane, an impurity of pentane (Figure 1(b)). n-Hexane co-eluted with TMSOH too (Figure 1(c)). Although the GC temperature program might be adjusted to resolve the coelution issues for n-pentane and n-hexane, the adjustment would require extra run time. Hence, n-decane was selected as the solvent for TMSOH analyses. This selection applies only to this specific column, but may be useful for other columns of similar properties.

#### 3.1.2. Effects of Desorption Temperature

**Figure 2:**
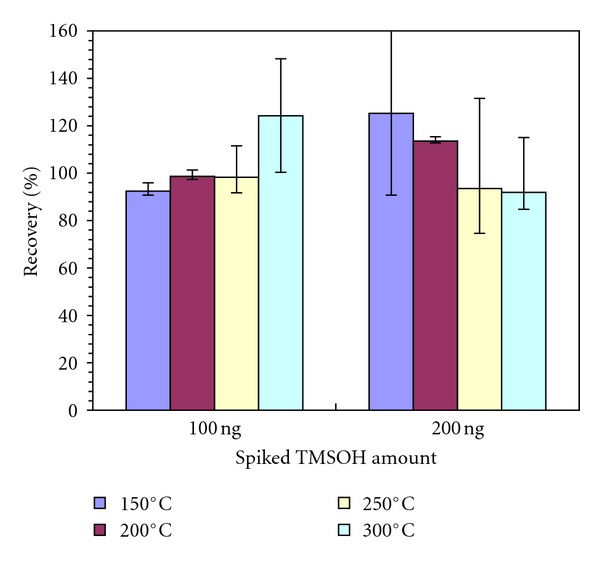
TMSOH abundances at different thermal desorption temperatures. Error bars show minimum and maximum recoveries.

The recoveries of TMSOH were similar at different thermal desorption temperatures, ranging from 92 to 125% (Figure 2). Consistent recoveries were expected as TMSOH is a highly volatile compound with a vapor pressure of 74 mmHg. High recoveries occurred at 300°C for a 100 ng loading and 150°C for a 200 ng loading. The results suggested that any desorption temperature between 150 and 300°C applies to TMSOH. The final method adopted 300°C in order to accommodate other less volatile compounds, for example, naphthalene and heavy alkanes.

#### 3.1.3. Effects of Adsorbent Configurations

The recoveries were 87 ± 15%, 87 ± 11%, and 33 ± 5% for single-, dual- and triple-bed tubes, respectively. Single- and dual-bed Tenax tubes had similar desorption efficiencies. Although Tenax tubes had no memory effects, dual-bed tubes were expected to have stronger “resistance” to breakthrough and ability to capture more volatile chemicals, due mainly to the high surface area of Carboxen 569. The recovery from triple-bed tube was poor. The second desorption of triple-bed tubes obtained 23–64% of amounts from the first desorption, indicating the memory effect that was related to the strong affiliation ability of Carbosieve-SIII in triple-bed tubes. As dual-bed tubes had potential to trap other more volatile species and to improve the accuracy and precision [31], they were selected as the sampling device.

In summary, the sampling and analytical method was optimized if using dual-bed tubes as sampling device, n-decane as the analysis solvent, and a desorption temperature of 300°C. The performance of the method was then evaluated using these parameters.

### 3.2. Laboratory Performance

#### 3.2.1. Retention Time

The retention time of TMSOH under the optimized GC condition was 5.143 min with a narrow range from 5.116 to 5.183 min.

#### 3.2.2. Recovery

The average recoveries were 87% (range 78–96%) and 87% (range 76–99%) at 100 and 200 ng loadings, respectively. The recovery increased to 126% (range 110–142%) when tubes were spiked with 5 ng of TMSOH. The higher recovery might be due to the omission of the IS in recovery tests.

#### 3.2.3. Precision

The replicate precision, expressed as relative standard deviation or percent difference, averaged 17.7% over a wide amount range from 0.1 to 500 ng. The precision deteriorated at lower spiked amounts, as reported for other VOCs [26, 32]. Thus, the average precision was 12.3% if lower spiked amounts (≤1 ng) were not considered. The relationship between precision and concentration could be modeled as:

(5) $$\begin{array}{c} \text{ln(Precision)}=-0.12\;\text{ln(Concentration)}+3.0. \end{array}$$

The negative coefficient clearly indicated the discordant relationship, although the association was medium (*R* ^2^ = 0.38) due to a small number of data points. Such a model can be used to evaluate reproducibility as well as other applications, for example, data imputation [32] and source apportionment models [33].

#### 3.2.4. Linearity

The 7-point calibration curves were determined as the following.

(6) With  IS: ATMSOHAIS=0.03042(MTMSOHMIS)+0.06042;

(7) without  IS: ATMSOH=32985MTMSOH+47791,

where *A* = Abundance and *M* = Amount (ng). The calibration curve expressed as (6) showed excellent linearity (*R* ^2^ = 0.9999) in the range of 0.1–500 ng of TMSOH in the SIM mode analysis. Linearity altered slightly if using the logarithm data (*R* ^2^ = 0.9970). Considering a nominal sample volume of 20 L used in the field, the method had a good linearity in the concentration range from 0.005 to 25 *μ*g/m^3^. It was also noted that the IS abundances were constant among analyses, displaying a fluctuation of less than 20%. The stability of the instrument suggested that a calibration could be established without an internal standard. Thus, a calibration curve was then constructed based solely on TMSOH abundance and mass (i.e., without using IS), formulated as (7). This calibration also displayed excellent linearity: *R* ^2^ = 0.9997 for untransformed data and *R* ^2^ = 0.9914 for log-transformed data. Not using internal standards has two advantages. First, the operations are simplified as tubes do not need to undergo the IS spiking step; second, it will be easier to capture other very volatile compounds that may co-elute with the solvent, for example, 1,3-butadiene, as no solvent is introduced. Such practice actually is not uncommon in environmental analyses. For these reasons, quantitation of TMSOH in field samples used (7), and the parameters were updated by performing a full calibration right before the laboratory analysis.

#### 3.2.5. Method Detection Limit

**Figure 3:**
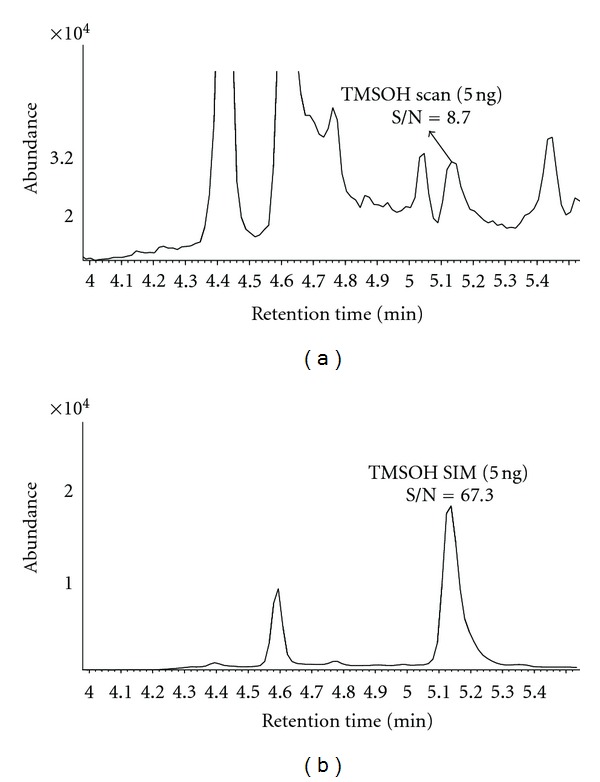
Total ion chromatograms showing signal-to-noise (S/N) ratios obtained from thermal desorption followed by GC-MS analysis of 5 ng of TMSOH in (a) MS scan mode and (b) MS SIM mode.

The analyses of seven 0.1 ng of TMSOH replicates yielded an MDL of 0.057 ng in SIM mode. This corresponded to MDLs of 2.8 and 9.5 ng/m^3^ for sample volumes of 20 L and 6 L, respectively. These MDLs were at least 100 times lower than the minimum concentration (1.0 *μ*g/m^3^) encountered in the field, as presented later. The low MDL took the advantage of the MS SIM mode. A comparison of signal-to-noise ratios between SIM and scan modes showed a 7.7 times higher sensitivity in SIM (Figure 3). This increment agreed with our previous findings, that is, 1.1- to 22-fold improvement of MDLs in SIM mode compared to those in scan mode [26].

#### 3.2.6. Retainability of TMSOH on Adsorbent(s)

Results of retainability tests were summarized in Table 2. The breakthrough of TMSOH in dual-bed tubes was negligible: the amounts of TMSOH in the backup tubes were mostly less than 2%, even at high loadings and/or flushing volumes. In contrast, significant portions (at least 20%) of TMSOH broke through the single bed Tenax, indicating poor retainability. Although not an exact mimic of the real-world sampling, the tests confirmed that dual-bed tubes were more suitable for TMSOH sampling with little chance of breakthrough while maintaining satisfactory recovery.

#### 3.2.7. Storage Stability

The loss of TMSOH in spiked sorbent tubes was 13% of the initial loading within 3 days. However, storage caused a larger loss of 23% for one week, and no further loss was observed afterwards. A previous study showed that the loss averaged 14% after 1-week storage for 51 compounds, and it was negligible from 1 week to upto 6 weeks [23]. Our tests showed similar decay pattern, while a faster decay was expected given the high volatility of TMSOH. The decay constant was −0.0401 day^−1^, similar to those of some compounds on Tenax adsorbents [26]. This decay constant indicates a half-life of 17.3 days for TMSOH on dual-bed adsorbents. A criterion of <15% loss means that TMSOH samples should be analyzed within 4 days.

### 3.3. Field Study

**Figure 4:**
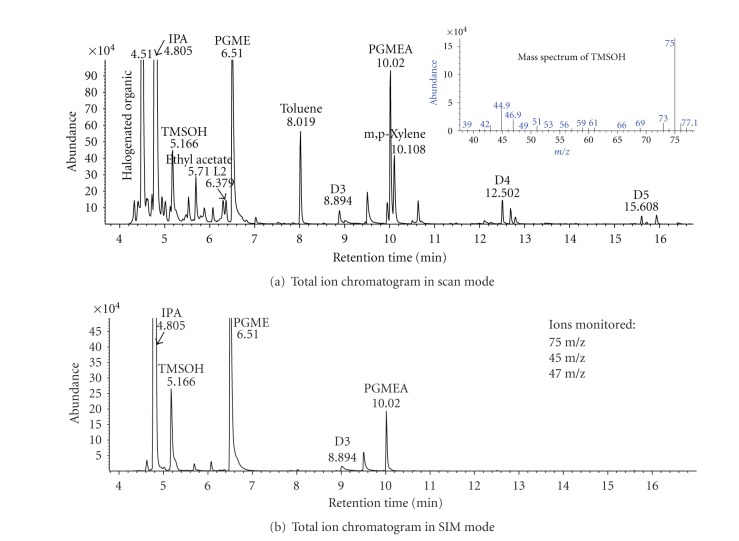
Total ion chromatograms of a typical field sample collected in a semiconductor fabrication workshop. Notes: IPA: Isopropyl alcohol; PGME: Propylene glycol methyl ether; PGMEA: Propylene glycol monomethyl ether acetate; L2: Hexamethyldisiloxane; D3: Hexamethylcyclotrisiloxane; D4: Octamethylcyclotetrasiloxane; D5: Decamethylcyclopentasiloxane.

Results of TMSOH samples collected in a semiconductor factory were summarized in Table 3, and the scan and SIM total ion chromatograms of a typical field sample were displayed in Figure 4. In the 2010 sampling campaign, TMSOH concentration was 2.58 *μ*g/m^3^ on average in the workshop, with a limited range from 1.02 to 5.98 *μ*g/m^3^. The follow-up sampling revealed much higher concentrations of 20.19–27.30 *μ*g/m^3^, mainly due to the higher wafer production in 2011. Even these limited data showed large spatial and temporal variation in airborne TMSOH in the workshop, possibly caused by the proximity of sources, the changes in the manufacturing process, ventilation conditions, and types of chemical air filters. The duplicate precision was 10.9% for distributed volume samples and 13.7% for same volume samples. These numbers were very close to what was observed in the laboratory. In summary, the method had a ppt (part-per-trillion) levels of MDL, which were sensitive enough to detect ppb (part-per-billion) levels of TMSOH in the field.

### 3.4. Limitations and Future Studies

While both the laboratory and field tests showed satisfactory performance for measuring TMSOH, this study had several limitations. The quantification was limited to only TMSOH, which was the interest and/or concern from the manufacturing perspective. The chromatograms of field samples revealed several other siloxane compounds, including L2 and D3–D5 siloxanes (Figure 4(a)), as well as many other common VOCs, such as benzene, toluene, ethyl benzene, xylenes, ethyl acetate, heptane, butyl ester, benzyl alcohol, octadecane, and hexadecane. The current GC conditions showed excellent separation of siloxanes, and thus had potential of monitoring a wide range of siloxanes, if needed. Stainless steel tubes may be another option for monitoring siloxanes, considering the potential contamination and surface reactions in glass tubes. The field data were limited for investigating the temporal and spatial variations and measurement uncertainty. Although our methods could obtain low enough MDLs within 60 min sampling, extra time is required for shipping, storage, and laboratory analyses. Thus, we want to highlight the need for developing real-time monitoring technique, one of the most active trends in VOC measurement instrumentation [34]. Such techniques will provide instantaneous and frequent readings of TMSOH that cannot be captured in conventional time-integrated sampling, and allow better product quality control in manufacturing. An online GC/FTIR system has been developed to measure ppm levels of TMSOH in biogas [35], but the sensitivity is not satisfactory for sub-ppb level encountered in semiconductor factories. Still, our methods will serve a technical basis for developing future miniature instruments, which often evolve enrichment of trace levels VOCs using adsorbents [36].

## 4. Conclusions

In this study, we developed a sensitive sampling and analytical method for measuring trace levels of TMSOH in indoor air of semiconductor fabrication environments. Method optimization suggested that best performance could be obtained if using dual-bed (Tenax TA followed by Carboxen 569) adsorbent configuration, n-decane as analysis solvent, and a thermal desorption temperature of 300°C. Laboratory and field evaluation revealed satisfactory performance of the methods: a reasonable recovery of 87%, typical replicate precision of within 15%, high linearity (*R* ^2^ = 0.9999), and a low MDL of 2.8 ng/m^3^ for a 20-L sample. The TMSOH on adsorbents could stay stable for up to 4 days, with a loss of 23% in 14 days and longer. TMSOH concentrations varied from 1.02 to 27.30 *μ*g/m^3^ in the indoor air of a semiconductor fabrication workshop. To our knowledge, these were the first measurements of indoor airborne TMSOH in occupational indoor air. We also suggest the need to develop real-time monitoring techniques for the maintenance and control of the scanner lens system during lithography process in semiconductor wafer manufacturing. 

## Figures and Tables

**Table 1:** Thermal desorption (TD)-GC-MS conditions.

TD parameters	
TD model	Gerstel thermal desorption system (TDS)/cooled injection system (CIS)
1st desorption temperature (°C)	300
1st desorption holding time (min)	5
1st desorption flow rate (mL/min)	80
Transfer line temperature (°C)	280
CIS cryofocusing temperature (°C)	−30
CIS 2nd desorption temperature (°C)	300
2nd desorption holding time (min)	5
Cryofocusing liquid	Liquid nitrogen (N_2_)

GC parameters	
GC model	Agilent 7890A GC
Split ratio	20 : 1
Column	HP-5MS (60 m Length, 0.25 mm I.D., 0.250 *μ*m film thickness)
Oven temperature program	40°C, hold for 2 min
	8°C/min to 180°C, hold for 2 min
	10°C/min to 250°C, hold for 1 min
	15°C/min to 300°C, hold for 5 min
Run time (min)	37.83

MS parameters	
MS model	Agilent 5975C inert XL MSD with triple-axis detector
Electron ionization voltage (eV)	70
Quadrupole temperature (°C)	150
Source temperature (°C)	230
Mass mode	Full scan and SIM (selected-ion monitoring),
Mass range (m/z)	35~550 in full scan
	45, 47, and 75 in SIM (for TMSOH)

**Table 2:** Results of retainability tests. Front, Back1, and Back2 were the front, 1st backup and 2nd backup tubes in series. Amounts of TMSOH in three tubes were expressed as the percentages of the initial amount spiked to the front tube.

Amount (ng)	Vol (L)	Flow matrix^a^	Dual-bed tubes			Single-bed tubes		
			Front (%)	Back1 (%)	Back2 (%)	Front (%)	Back1 (%)	Back2 (%)
10	1	N_2_	98.2	1.1	0.7	91.6	4.4	3.9
10	5	N_2_	96.0	0.7	3.3	66.6	28.7	4.8
10	10	N_2_	99.4	0.4	0.2	78.7	20.8	0.5
10	20	N_2_	97.8	1.0	1.2	68.5	27.1	4.5
200	1	N_2_	99.5	0.5	0.1	94.3	5.7	0.0
200	5	N_2_	98.9	0.3	0.8	24.6	34.0	41.4
200	10	N_2_	97.8	1.1	1.2	18.4	35.0	46.7
200	20	N_2_	99.5	0.4	0.1	23.0	38.5	38.5
200	20	Air	99.7	0.3	n.a.	35.8	64.2	n.a.
200	20	Air	98.4	1.6	n.a.	40.9	59.1	n.a.

Notes: ^a^Flow matrix: the gas blown through tubes in retainability tests. n.a.: not available.

**Table 3:** TMSOH concentrations measured in a semiconductor fabrication workshop.

Sampling date	Sample volume (L)	TMSOH concentration (*μ*g/m^3^)	
		Rep 1	Rep 2
06/23/2010	6	1.58	n.a.
07/08/2010	6	1.32	n.a.
07/21/2010	6^a^	1.21	n.a.
	20^a^	2.82	n.a.
08/02/2010	6	5.98	n.a.
08/25/2010	20^a^	1.02	n.a.
	6^a^	2.61	n.a.
09/30/2010	6	3.91	n.a.
10/14/2010	20	2.74	n.a.
08/25/2011	6/20^b^	22.51	20.19
08/26/2011	6^c^	23.80	27.30

Notes: ^a^Samples at two locations within the same workshop. ^b^Co-located distributed volume replicate samples, ^c^Co-located same volume replicate samples. n.a.: not available.
